# Immune System Modeling and Analysis

**DOI:** 10.3389/fimmu.2014.00644

**Published:** 2014-12-19

**Authors:** Ramit Mehr

**Affiliations:** ^1^Computational Immunology Lab, The Mina and Everard Goodman Faculty of Life Sciences, Bar-Ilan University, Ramat-Gan, Israel

**Keywords:** immune system, mathematical modeling, lymphocytes, repertoire, immunomics

Immunologists currently face daunting challenges, as a result of the rapid development of new methods for immunological data collection, from high-throughput phenotyping to deep sequencing (1). These and similar methods keep generating humongous amounts of immunological data, which in turn challenge the theoretical immunology community to develop methods for data organization and analysis and mathematical and computational modeling. These challenges and methods were discussed in recent workshops, for example the Lymphocyte Repertoire Workshop (Institute of Advanced Studies of the Hebrew University, Jerusalem, early 2012, organized by myself), and the International Seminar on Multi-Scale Physics of Lymphocyte Development (Max Planck Institute for the Physics of Complex Systems, Dresden, Summer 2012, organized by M. Or-Guil et al.).

At about the same time, the organizers mentioned above were approached by the Frontiers editorial staff with the idea for a “Frontiers in Immunology” research topic, which was to provide a comprehensive, online, open access snapshot of the current state of the art on immune system modeling and analysis. The research topic was launched, edited, and finalized with the kind help of co-editors Rob de Boer, Miles Davenport, Carmen Molina-Paris, Michal Or-Guil, and Veronika Zarnitsyna. It has been a success, with 35 papers accepted for publication, which attests to the timeliness of the topic.

The papers included in this Research Topic reflect many of the issues that theoretical immunologists are struggling with. Some of the papers address old questions – such as the targeting of somatic hypermutation (2) and the resulting diversity of B cell repertoires (3, 4), how clonal selection operates in germinal centers (5–8); or how the T cell compartment develops (9–11) and changes with aging (12). However, these papers offer new viewpoints, which emerged thanks to the immunological “data revolution”, in particular next-generation sequencing of lymphocyte repertoires. Others address new methods of extracting (13–15) and analyzing (16–18) comprehensive T and B cell phenotype and repertoire data, and delineate some of the first insights gleaned from sequencing studies regarding how these repertoires emerge, evolve, and function (19–25). Natural killer cells (26), myeloid cells (27), and structural immunology (28–31) are also represented.

My thanks go to the above-mentioned co-editors, to the responsive and efficient Frontiers editorial staff, to all the authors who contributed papers, and to the reviewers whose work has made publication of all these papers possible.

## Conflict of Interest Statement

The author declares that the research was conducted in the absence of any commercial or financial relationships that could be construed as a potential conflict of interest.
